# Balancing flexibility and ethical oversight: Japan’s regulatory framework for human stem cell-based embryo model research

**DOI:** 10.1016/j.stemcr.2026.103085

**Published:** 2026-09-24

**Authors:** Ayako Kamisato, Hidenori Akutsu

**Affiliations:** 1 Department of Medical Legal Research, National Center for Child Health and Development, Tokyo, Japan; 2 Center for Regenerative Medicine, National Center for Child Health and Development, Tokyo, Japan

## Abstract

Japan revised its national government guidelines in 2026 to regulate research on human stem cell-based embryo models (SCBEMs). Based on the view that current SCBEMs are distinct from human embryos, the framework adopts relatively flexible oversight while prohibiting reproductive use and allowing future revision as scientific advances emerge.

## Introduction

Stem cell-based embryo models (SCBEMs) provide a new experimental platform for investigating pre- and post-implantation development, cell lineage determination, morphogenesis, early pregnancy abnormalities, and the evaluation of drugs and environmental factors (Wu and Wang, 2026). Patient-derived, disease-specific SCBEMs are useful for elucidating pathological mechanisms (Rosner et al., 2025). The importance of research involving the creation and use of SCBEMs (“SCBEMs research”) is expected to continue to grow.

SCBEMs are expected to circumvent ethical concerns because they are derived from stem cells rather than fertilized embryos and can be generated without destroying embryos. However, if SCBEMs that are morphologically and functionally indistinguishable from human embryos, or that develop into a human individual, are created in the future, should we grant them the same moral status as human embryos and apply equivalent regulations? Even if SCBEMs do not acquire such developmental potential, to what extent will society tolerate the research use of models that exhibit features relevant to individuality or moral status, such as nervous system development, body-axis formation, primitive streak formation, or the capacity to generate germ cells? SCBEMs present us with new ethical challenges that must be addressed. In response, the International Society for Stem Cell Research (ISSCR) added provisions regarding SCBEMs in the 2021 revision of its “Guidelines for Stem Cell Research and Clinical Translation”; specifically, it introduced “integrated” and “non-integrated” embryo models, stipulating that transfer of either model to the uterus of a living human or animal host is prohibited; that the former must be reviewed by a specialized oversight process; and that culturing must be limited to the minimum period necessary to achieve the scientific objectives (International Society for Stem Cell Research, 2021). Furthermore, in 2025, a targeted update addressing significant advances in the development and application of SCBEMs was published, including a replacement of embryo model classification with the inclusive term “SCBEMs”; an explicit ban on ectogenesis; and an expansion of the scope of the subject to specialized oversight processes for all 3D SCBEMs (International Society for Stem Cell Research, 2025).

While discussions on SCBEMs regulation are progressing in various countries, only a limited number have established explicit regulatory frameworks through laws or government guidelines (Arias et al., 2026). In Japan, government guidelines were revised in 2026, making SCBEMs research explicitly subject to regulation. The authors were involved in developing this regulatory framework. Fujita et al. recently described the scope of the revised Japanese guidelines, their relationship to the ISSCR framework, and the associated operational challenges (Fujita et al., 2026). Complementing that account, this forum focuses on the historical and ethical reasoning underlying Japan’s regulatory choices and on how the framework is intended to evolve as the science advances.

## Perspectives on human embryos and ethical principles for human embryo research in Japan

To understand the regulations governing SCBEMs research, one must first understand the regulatory framework for human embryo research in Japan.

In 2001, an Expert Panel on Bioethics (Panel) was established under the Council for Science and Technology Policy of the Japanese Cabinet Office. The Council published the report “Basic Principles on the Handling of Human Embryos” in 2004, based on the Panel’s opinions (Council for Science and Technology Policy, 2004). It defined the human embryo, including a cloned embryo, as—while not a “person”— the “germ of human life” because if situated within the maternal body, it can develop into a fetus and be born as a “person.” Consequently, an embryo was deemed an entity that “must be accorded special respect in order to uphold the fundamental societal value of “human dignity.” Therefore, in general, the conduct of research that damages human embryos or the creation of human embryos for research purposes was considered unacceptable. However, the pursuit of well-being regarding human health and welfare was also deemed to be grounded in fundamental human rights. From this perspective, the report made exceptions: the creation and use of human embryos were permissible if they had the potential to bring scientific or medical benefits that were both scientifically rational and socially justifiable, and were reviewed by a research ethics committee (REC) and the national government. These constitute ethical principles regarding human embryo research in Japan. Regulations have been developed based on these principles, through a process in which the Panel deliberated and prepared reports, and the relevant government formulated or revised guidelines based on the Panel’s reports.

## The process and background of SCBEMs research regulation in Japan

In April 2023, the Panel decided to deliberate on SCBEM research. Subsequently, a working group (WG) was established under the Panel and submitted its report to the Panel in April 2024 (Working group for the examination of creating human embryo-like structures from pluripotent stem cells, 2024). The Panel conducted further deliberations and compiled the Panel report, Handling of Human Stem Cell-Based Embryo Models (Interim Summary),” in November 2024 (Expert Panel on Bioethics, 2024). The following is an overview of the Panel report, which laid the foundation for SCBEMs research regulations in Japan (Sawai et al., 2025).

The Panel report defined “human embryo models” as structures resembling human embryos created from human embryonic stem (ES) cells, human induced pluripotent stem (iPS) cells, and human tissue stem cells other than germ cells. In this forum, to align with international terminology, these are referred to as SCBEMs. At the time, the ISSCR guidelines classified SCBEMs into two types. However, the Panel decided not to adopt this classification because the boundary between the two is ambiguous, SCBEMs under study are diverse, and further subdivisions are likely to occur in the future.

The Panel first examined the differences between human fertilized embryos and SCBEMs. It concluded that, at this stage, SCBEMs currently remain cell aggregates that exhibit only some features of embryos at the blastocyst, implantation, or later stages. Since such SCBEMs cannot be born as human beings, even if transplanted into a mother’s womb (as inferred from experiments using mouse SCBEMs), they are not equivalent to human fertilized embryos. Next, the Panel examined whether cloned embryos and SCBEMs might share similar characteristics. The “Act on Regulation of Human Cloning Techniques,” enacted in 2000, prohibits the creation of cloned humans possessing the same genetic material to preserve human dignity and imposes strict conditions on the creation of human cloned embryos. If human ES cells are established from a human cloned embryo to create SCBEMs, or if multiple SCBEMs are created from the same human iPS cells, ethical concerns similar to those regarding human cloned embryos may arise. However, because current SCBEMs are not reasonably expected to develop into a human individual, even if transferred into a human or animal womb, the Panel concluded that regulations equivalent to those for human cloned embryos are unnecessary.

At the same time, the Panel recognized that advances in science and technology may lead to SCBEMs with developmental properties comparable to those of human embryos, or with a high degree of embryo-like organization. It also acknowledged the possibility that future SCBEMs may exhibit features relevant to individuality, moral status, or human identity. Furthermore, concerns were raised about inappropriate research practices, including the transfer of SCBEMs into a human or animal womb. Therefore, the Panel decided to regulate SCBEMs by considering the current progress of SCBEMs research. “Interim Summary” in the report title indicates that the regulatory framework will be re-examined as research progresses further and SCBEMs approach the same level of protection as human embryos. This approach is conceptually consistent with the “tipping-point” framework proposed by Rivron et al., under which increasing moral significance may warrant progressively stronger oversight (Rivron et al., 2023).

At this stage, the Panel decided to prohibit the transfer of SCBEMs into a human or animal womb; require REC “review” and “notification” to the national government, not reviewed by it, for all SCBEMs research; for SCBEMs research using existing cell stocks, which were non-ES cell stocks, as with general clinical research, opt-out procedures might be permitted when obtaining consent was impracticable or the research was conducted for academic purposes. The latter two policies were more relaxed than those for human embryo research to balance the potential benefits of SCBEMs research. The Panel also emphasized the social acceptance of SCBEMs research, requesting research institutions to make their research findings public, and researchers to strive to promote public understanding through outreach and education.

Regarding the “culture period,” the WG report took a stance similar to that of the Nuffield Council on Bioethics report, which also argued that an upper limit should be set (Nuffield Council on Bioethics, 2024). However, the Panel revised this position, as the emergence of SCBEMs mimicking human morphology or fetus-like structures with developed brain and neural functions was considered implausible at this stage. This policy change means Japan has adopted one that prioritizes scientific feasibility and research flexibility (Table 1).

**Table 1:** Japan’s regulatory policy as determined by the Panel report

Review system	Unlike single-tier systems that rely solely on ethical review, Japan adopts two-tier systems that require both REC review and notification to the national government all SCBEMs research, including research that is typically exempt from ethical review (e.g., using commercially available cells), shall be reviewed by a REC established in accordance with the guidelines; the research is required to notify the national government, but since SCBEMs are not human embryos, it does not require government review
Prohibited activity	Implanting SCBEMs into the wombs of humans or animals shall be prohibited, since there is no scientific justification and such practices are ethically unacceptable; given the potential for future advancements in research on artificial wombs and similar technologies, it is necessary to establish a prohibition that covers all research leading to human reproduction
Culture period	Researchers shall set the minimum culture period necessary to achieve the research objectives in the research protocol, and the REC shall review its appropriateness
Promoting public understanding	Research institutions shall make their research findings public; researchers shall take every opportunity to provide information about their research and strive to promote public understanding through outreach and education
Informed consent	For non-ES stem cell-based SCBEMs research, the guidelines do not impose a uniform informed-consent requirement equivalent to that for germ-cell generation research; opt-out procedures may be permitted when existing cell stocks are used, obtaining consent is impracticable, or the research falls within the scope of academic research, in accordance with “ethical guidelines for medical and biological research involving human subjects”

## Regulations on SCBEMs research that have been introduced

Following this Panel report, the Ministry of Education, Culture, Sports, Science, and Technology (MEXT) discussed how to incorporate the policies outlined in Table 1 into existing relevant guidelines. Accordingly, the “Guidelines on the Utilization of Human ES cells” and the “Guidelines on the Research on Producing Germ Cells from Human iPS Cells or Human Tissue Stem Cells” were revised to regulate SCBEMs research using human ES and iPS cells or other stem cells, respectively. The latter guideline was also renamed the “Guidelines on the Research on Producing Germ Cells or Human Embryo Models from Human iPS Cells or Human Tissue Stem Cells” (iPS Guidelines). These were promulgated on February 13 and came into effect on April 1, 2026.

A definition of SCBEMs was added to these guidelines. Specifically, it was defined as “a group of cells derived from differentiated human stem cells exhibiting characteristics of human embryos or early-stage embryonic cell clusters similar to human embryos but are not human embryos.” Unlike the ISSCR 2025 Guidelines, which emphasize three-dimensional (3D) spatially organized human SCBEMs as a key category requiring specialized oversight, the Japanese guidelines adopt a functional criterion. This approach involves evaluating the possibility that SCBEMs have acquired functions associated with the moral characteristics of human embryos.

The guidelines implement the policies in Table 1 as follows. New provisions regarding “prohibited activities” and “promoting public understanding” were added to both guidelines. Regarding “informed consent,” the iPS Guidelines deliberately refrained from establishing a provision that makes the obtaining of informed consent a mandatory requirement for SCBEMs research. For research using ES cells, additional provisions were deemed unnecessary because the embryo donors provide informed consent at the time of ES cell line establishment, including consent for the use of the resulting ES cell line in a broad range of research. “Culture period” was added as a mandatory item in research protocols, and researchers were responsible for explaining the rationale for the minimum period. Regarding “review system,” SCBEMs research was put under the two-tier oversight system: reviewed by an REC established under the guidelines and subsequent notification to MEXT. This system facilitates the rapid initiation of research; however, the REC shall assess, in line with the latest scientific knowledge, whether the objective could be achieved using a shorter period or a less developmentally complete model (Rivron et al., 2023; Sugarman, 2025), whether SCBEMs have the potential to develop into individuals or acquire functions associated with the moral characteristics of human embryos. To make these determinations, the REC needs a high-level reviewing capability. Therefore, the government should provide support—such as identifying the morally relevant features of SCBEMs to consider during review (Moris et al., 2026); establishing a system to dispatch experts to the REC—to enable the REC to conduct appropriate reviews. The guidelines set provisions of procedures for SCBEMs transfer, allowing their transfer to overseas research institutions to conduct international collaborative research under appropriate oversight. Furthermore, both guidelines impose a reporting obligation on researchers, ensuring consistency with the 2025 ISSCR Guidelines’ recommendation (Recommendation 2.2.2.2).

## Conclusion

Japan has established a relatively flexible regulatory framework for SCBEMs research, with particular significance in establishing a system that enables the national government to monitor the implementation of SCBEMs research through a “notification” process. As SCBEMs research continues to advance, new issues are likely to arise. Monitoring research trends enables the identification of ethical issues that need to be discussed and of flexible regulatory revisions.

Japan’s experience will serve as a reference case for common challenges in regulatory design for rapidly advancing science and technology, namely, balancing flexibility and consistency, especially for countries where discussions on the regulation of SCBEMs research are ongoing.

## Acknowledgments

This research was supported by 10.13039/100009619AMED under grant numbers JP25oa0439002 and JP25bm1323001h0303.

## Author contributions

A.K. led the drafting of the manuscript. H.A. contributed substantive comments throughout the drafting process.

## Declaration of interests

The authors declare no competing interests.

## Declaration of generative AI and AI-assisted technologies in the writing process

This manuscript was drafted using DeepL for initial language assistance. The text was then comprehensively reviewed and edited by Editage (https://www.editage.jp) to ensure academic clarity and fluency. The authors take full responsibility for the content and integrity of the work.
